# A standardized extract of *Asparagus officinalis* stem improves HSP70-mediated redox balance and cell functions in bovine cumulus-granulosa cells

**DOI:** 10.1038/s41598-021-97632-6

**Published:** 2021-09-13

**Authors:** Khoi Thieu Ho, Kohei Homma, Jun Takanari, Hanako Bai, Manabu Kawahara, Khang Thi Kim Nguyen, Masashi Takahashi

**Affiliations:** 1 Graduate School of Agriculture, Hokkaido University, Sapporo, Hokkaido 060-8589 Japan; 2 AMINO UP Co. Ltd., Sapporo, Hokkaido Japan; 3 College of Agriculture, Can Tho University, Can Tho City, Vietnam; 4 Graduate School of Global Food Resources, Hokkaido University, Sapporo, Hokkaido 060-8589 Japan; 5 Research Faculty of Agriculture, Hokkaido University, Sapporo, Hokkaido 060-8589 Japan

**Keywords:** Cell biology, Molecular biology, Biomarkers, Medical research

## Abstract

Heat shock (HS) protein 70 (HSP70), a well-known HS-induced protein, acts as an intracellular chaperone to protect cells against stress conditions. Although HS induces HSP70 expression to confer stress resistance to cells, HS causes cell toxicity by increasing reactive oxygen species (ROS) levels. Recently, a standardized extract of *Asparagus officinalis* stem (EAS), produced from the byproduct of asparagus, has been shown to induce HSP70 expression without HS and regulate cellular redox balance in pheochromocytoma cells. However, the effects of EAS on reproductive cell function remain unknown. Here, we investigated the effect of EAS on HSP70 induction and oxidative redox balance in cultured bovine cumulus-granulosa (CG) cells. EAS significantly increased *HSP70* expression; however, no effect was observed on *HSP27* and *HSP90* under non-HS conditions. EAS decreased ROS generation and DNA damage and increased glutathione (GSH) synthesis under both non-HS and HS conditions. Moreover, EAS synergistically increased *HSP70* and *HSF1* expression and increased progesterone levels in CG cells. Treatment with an HSP70 inhibitor significantly decreased GSH level, increased ROS level, and decreased *HSF1*, *Nrf2*, and *Keap1* expression in the presence of EAS. Furthermore, EAS significantly increased progesterone synthesis. Thus, EAS improves HSP70-mediated redox balance and cell function in bovine CG cells.

## Introduction

Cumulus-granulosa (CG) cells surround oocytes and play a vital role in the maturation and acquisition of developmental competence in mammalian oocytes^1^. Cumulus cells and oocytes communicate metabolically via gap junctions, which provide important points of entry for nutrient transfer and signaling between both cell types^2^. The absence of CG cells has harmful effects on the maturation, fertilization, and embryo development in cattle^3^. CG cells enhance the nuclear and cytoplasmic maturation of oocytes during maturation^4^ and fertilization rate during fertilization^5^. In addition to playing an important role in oocyte development, CG cells protect oocytes against damage caused by oxidative stress during maturation^6^. After ovulation, the remaining granulosa cells differentiate into large luteal cells^7^. Bovine luteal cells produce progesterone (P4), an important steroid hormone, to maintain pregnancy^8^. Besides that, cumulus cells involved to P4 synthesis under in vitro condition^9^. In addition, the in vitro environment has a higher concentration of O_2_, which is a source of ROS, than the in vivo environment^10^. Cumulus cells have been suggested to play a critical role in defending bovine oocytes against cell damage due to ROS production^1^. Moreover, during in vitro maturation, cumulus–oocyte complexes exhibit higher levels of GSH than cumulus-denuded oocytes, and bovine CG cells contribute to cumulus–oocyte complex GSH synthesis^6^. Therefore, bovine CG cells play a major role in the generation of GSH, which can reduce ROS production. As a result, the balance between ROS and GSH levels can prevent cell death by maintaining the balance of the cellular redox status.

The cellular redox status contributes to important cellular functions, such as regulation of proliferation, differentiation, and cell death, and is defined as the balance between oxidants (or pro-oxidants) and antioxidants^11^. ROS, such as superoxide anion radicals, hydroxyl radicals, and hydrogen peroxide, are free radicals that cause damage to cells by lipid peroxidation and enzyme inactivation^12^. The upregulation of ROS production can induce cell death signaling through an imbalance in the redox status of the cells^13^. In addition, previous studies have evaluated the effect of ROS on DNA damage, which induces toxicity in cells or cell death^14^. In contrast, GSH, which is synthesized from the γ-glutamyl cycle, is one of the major antioxidants present in mammalian cells and provides a powerful antioxidant defense against oxidative stress^15^. The balance between ROS and GSH levels can reduce DNA damage by altering the cellular redox status, thus improving cell survival.

Heat shock (HS) protein 70 (HSP70), also called stress protein, is a molecular chaperone that assists in the folding, unfolding, and homeostasis of cellular proteins^16^. Therefore, the main functions of HSP70 allow the cell to survive during several stresses, such as physical, chemical, and environmental stresses^17^. Experimental evidence has suggested that HSP70 expression regulates both GSH and ROS generation, indicating an inter-relationship between HSP70 and the redox status^18^. Various plant sources have been studied to identify the HSP70 induction activity. In this context, asparagus (*Asparagus officinalis* L.) is a potential candidate because of its antioxidant ability^19^. In addition, functional food ingredients are more valuable when they promote the effective use of unused parts. EAS is produced from the unused bottom part of asparagus, and this extract contains active ingredients, such as asparagine, that enhance HSP70 expression^20^. In addition, asparagine has been found to increase HSP70 mRNA levels in a human promyelocytic leukemia cell line^20^. This evidence suggests that EAS is a potential inducer of HSP70, which may regulate the balance between GSH and ROS generation.

Several products, such as paeoniflorin, geranylgeranylacetone, and bimoclomol, have been found to stimulate HSP70^21,22^. Bimoclomol induces HSP expression under HS conditions but does not influence HSP activity under non-stress conditions^21^. Paeoniflorin and geranylgeranylacetone increased HSP70 expression under HS conditions in experiments on cells and rats^22^. These data showed that the HSP70 inducer has a synergistic effect under HS on *HSP70* expression.

In the current study, we investigated the effect of EAS on HSP70 induction and oxidative redox balance in cultured bovine CG cells.

## Results

### Effect of EAS on the specific induction of HSP70

**Figure 1:**
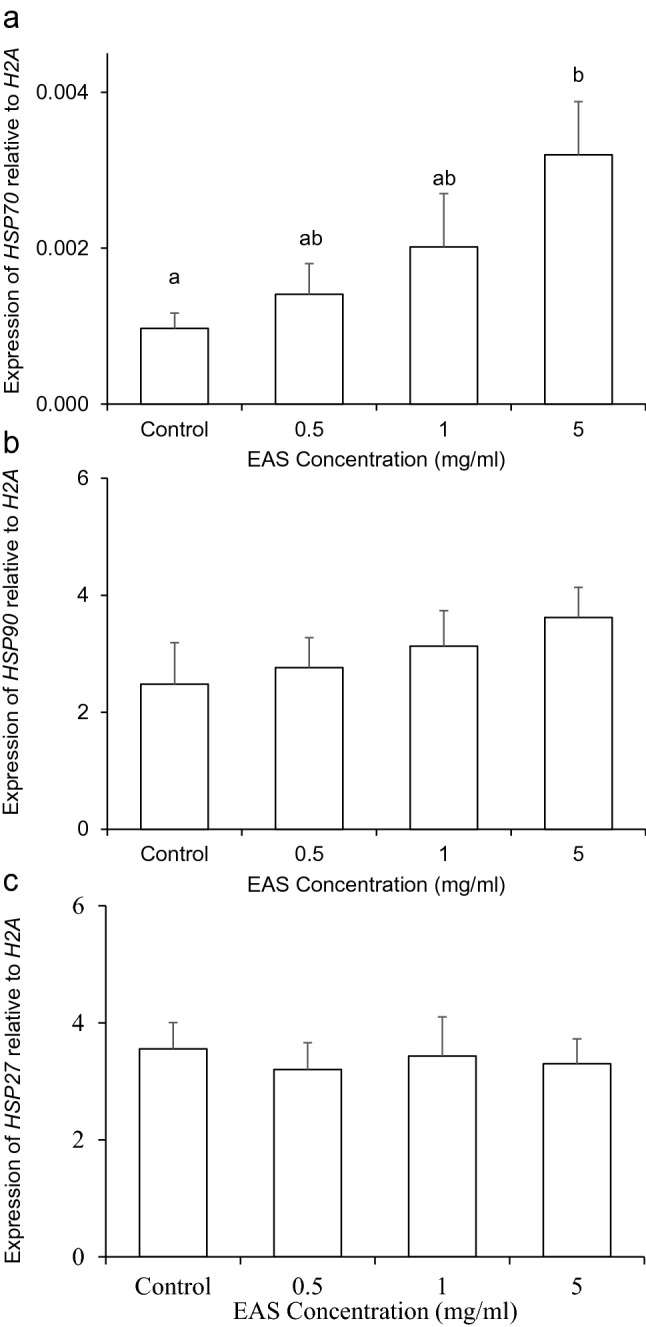
Effect of EAS concentration on the expression of *HSP70*, *HSP90*, and *HSP27* in bovine CG cells. Cells were treated with various concentrations of EAS (0.5, 1, and 5 mg/ml) for 6 h at 38.5 °C and analyzed for gene expression. The expression levels of *HSP70, HSP90, and HSP27* were examined using real-time quantitative PCR, normalized to *H2AFZ* as a reference gene. (**a**) *HSP70*, (**b**) *HSP90*, and (**c**) *HSP27*. Data are shown as the mean ± SEM, n = 5, a vs. b (*P* < 0.05).

**Figure 2:**
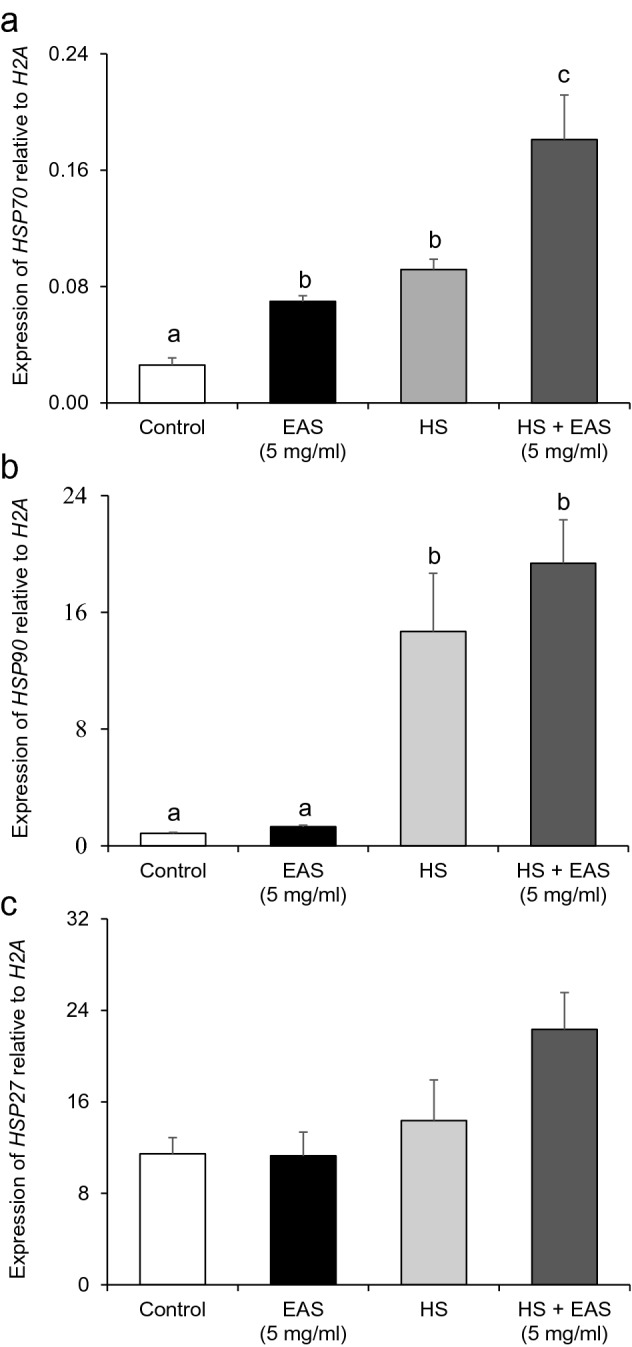
Effect of EAS on the expression of *HSP70, HSP90*, and *HSP27* in bovine CG cells under non- and HS conditions. Cells were treated for 6 h with or without EAS (5 mg/ml) under normal conditions at 38.5 °C (control, EAS group) and HS conditions at 41 °C (HS, HS + EAS group). (**a**) *HSP70*, (**b**) *HSP90*, and (**c**) *HSP27*. Data are shown as the mean ± SEM, n = 5, a vs. b (*P* < 0.05), a vs. c (*P* < 0.01), and b vs. c (*P* < 0.01).

To determine the concentration of EAS required to induce the expression of *HSP27*, *HSP70*, and *HSP90* in bovine CG cells, we examined various concentrations of EAS (0.5, 1, and 5 mg/mL). A significant increase (*P* < 0.05) in *HSP70* expression was observed after treatment with 5 mg/mL EAS (Fig. 1a). However, the expression of *HSP90* and *HSP27* was not influenced by EAS (Fig. 1b,c). Since *HSP*s are induced by HS, we evaluated the effect of EAS on HSP expression after 6 h of HS (41 °C) treatment in comparison with that under non-HS conditions in bovine CG cells. Similar to the findings shown in Fig. 1, EAS treatment specifically increased *HSP70* expression under non-HS control conditions (Fig. 2a). Moreover, in these experiments, the expression of *HSP90* (Fig. 2b) and *HSP27* (Fig. 2c) did not change.

**Figure 3:**
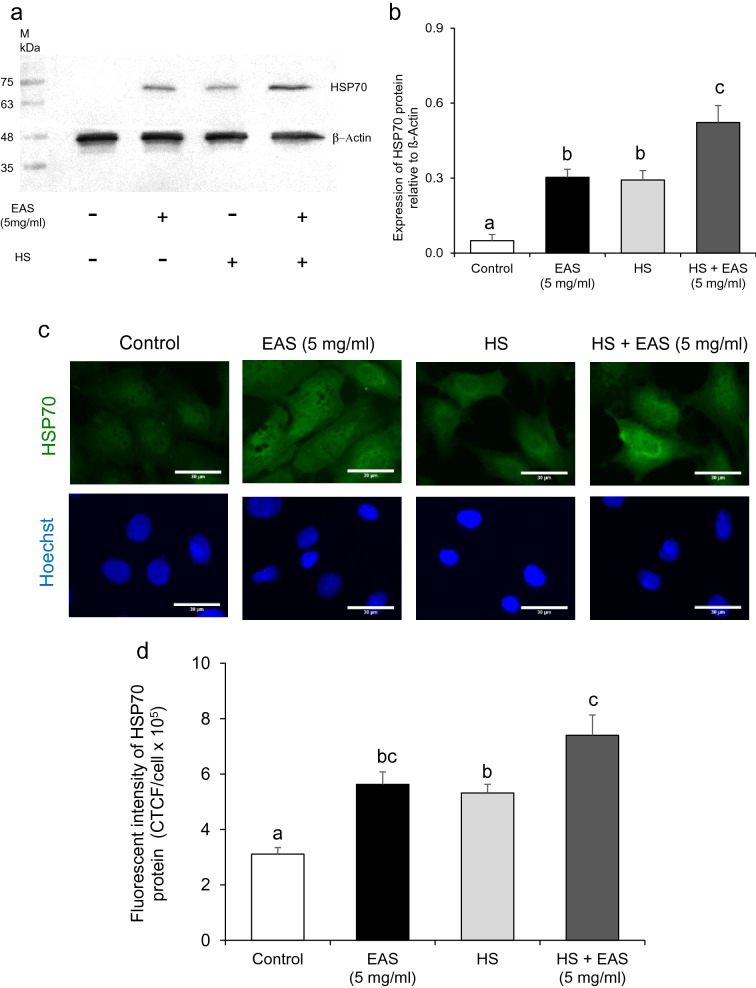
Effect of EAS on the expression of HSP70 protein in bovine CG cells. Cells were treated for 6 h with or without EAS (5 mg/ml) under normal conditions at 38.5 °C (control, EAS group) and HS conditions at 41 °C (HS, HS + EAS group). (**a**) Western blotting to elucidate the expression profile of HSP70 (upper bands) and β-actin (lower bands). Left lane shows the molecular marker from 35 to 75 kDa. (**b**) The expression level of HSP70 protein was normalized to that of β-actin. (**c**) Immunostaining for HSP70, Hoechst, and HSP70 Immunostaining images are shown at magnification × 40, and the scale bar is 30 μm. (**d**) Bars showing CTCF analysis of fluorescence intensity. (**a**) Data are shown as the mean ± SEM, n = 3, a vs. b (*P* < 0.05), a vs. c (*P* < 0.01), and b vs. c (*P* < 0.05). (**d**) Data are shown as the mean ± SEM, n = 5, a vs. b (*P* < 0.05), a vs. c (*P* < 0.01), and b vs. c (*P* < 0.05).

HS induced an increase in *HSP70* and *HSP90* expression. Interestingly, *HSP70* expression was synergistically increased by HS and EAS (Fig. 2). Similar to the induction of *HSP70*, HSP70 protein levels were significantly increased (*P* < 0.05) after treatment with 5 mg/mL EAS and synergistically increased (*P* < 0.01) by HS and EAS (Fig. 3a,b). The immunodetection of HSP70 also showed a similar increase in *HSP70* gene expression (Fig. 3c,d).

**Figure 4:**
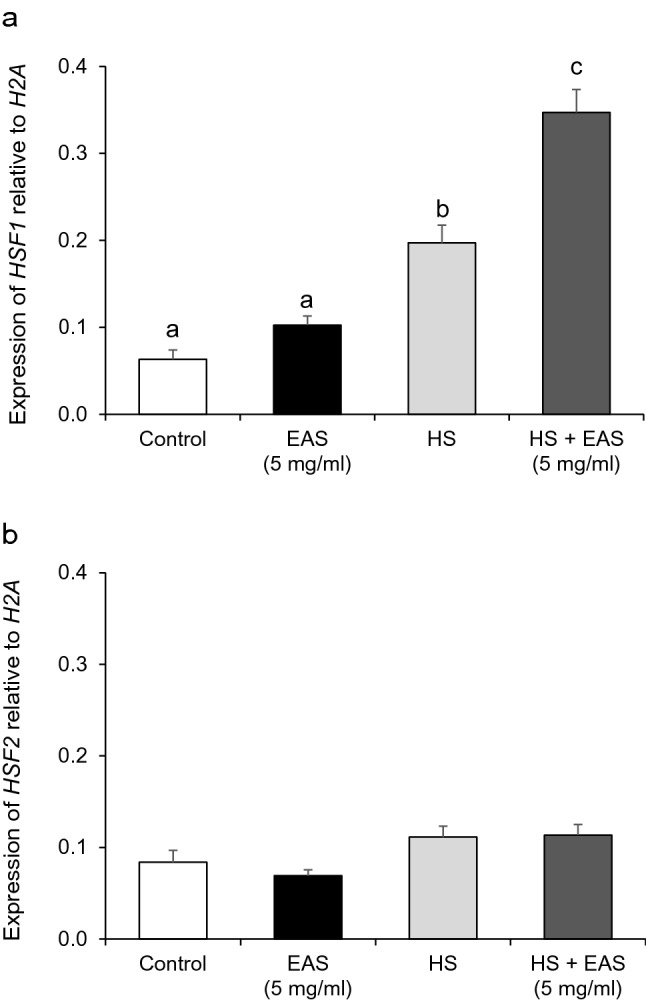
Effect of EAS on the expression of *HSF1* and *HSF2* in bovine CG cells under non- and HS conditions. Cells were treated for 6 h with or without EAS (5 mg/ml) under normal conditions at 38.5 °C (control, EAS group) and HS conditions at 41 °C (HS, HS + EAS group). The expression levels of (**a**) *HSF*1 and (**b**) *HSF2* were examined using real-time quantitative PCR normalized to *H2AFZ* as a reference gene. Data are shown as the mean ± SEM, n = 5, a vs. b (*P* < 0.05), a vs. c (*P* < 0.01), and b vs. c (*P* < 0.01).

HSF1 is a transcription factor that induces HSP70 expression by HS^23^. As shown in Fig. 4a, *HSF1* expression was significantly increased by HS and HS + EAS, whereas no effect of HS and HS + EAS on *HSF2* expression was observed (Fig. 4b).

### Effect of EAS on ROS generation and DNA damage

**Figure 5:**
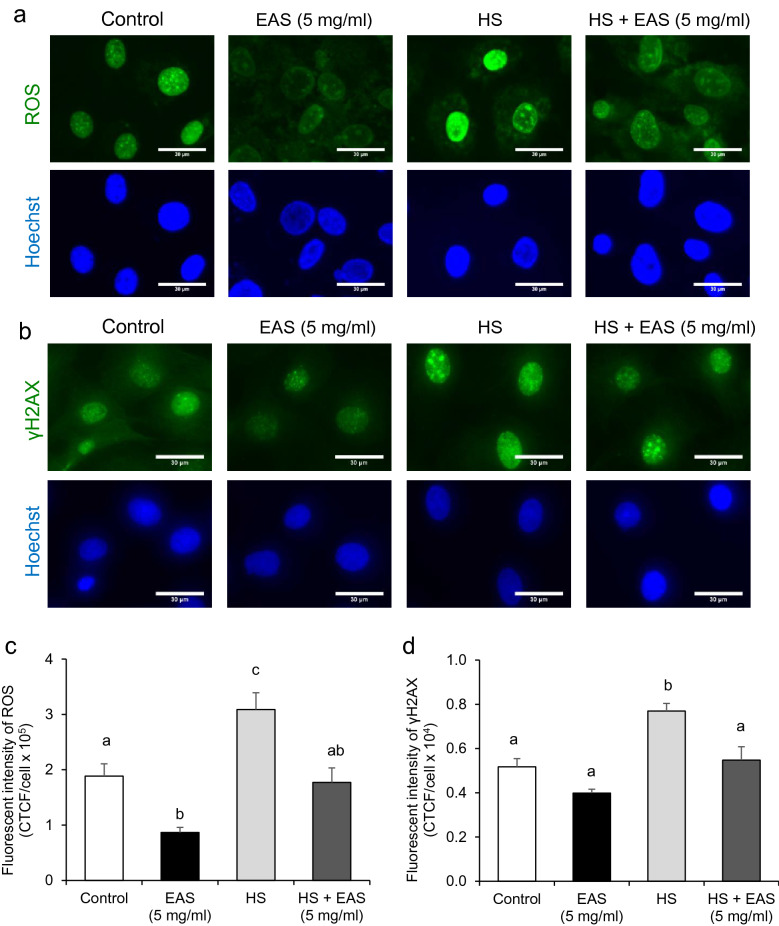
Effect of EAS on induction ROS and DNA damage stained γH2AX immunostaining in the bovine CG cells under non- and HS conditions. Bovine CG cells were treated for 6 h with or without EAS (5 mg/ml) under normal conditions at 38.5 °C (control, EAS group) and HS conditions at 41 °C (HS, HS + EAS group). (**a**) Fluorescence of ROS (upper) and nuclei stained with Hoechst (lower panel). (**b**) Immunostaining for γH2AX (upper panel) and nuclei stained with Hoechst (lower panel). Bar shows 30 μm. (**c**) Fluorescence intensity of ROS (CTCF). Bar shows 30 μm. (**d**) Fluorescence intensity of γH2AX (CTCF). (**c**) Data are shown as the mean ± SEM, n = 5, a vs. b (*P* < 0.05), a vs. c (*P* < 0.001), b vs. c (*P* < 0.05), and ab vs. c (*P* < 0.01). (**d**) Data are shown as the means ± SEM, n = 3, a vs b (*P* < 0.05).

After HSP70 expression was enhanced by EAS treatment in bovine CG cells, we investigated the effect of EAS on HSP70 expression and oxidative and redox balance^24^. As shown in Fig. 5a and c, EAS treatment significantly reduced ROS levels under both non-HS and HS conditions.

ROS are known to cause apoptosis associated with mitochondrial dysfunction and single and double-stranded DNA breaks^25^. Therefore, we hypothesized that EAS could reduce DNA damage caused by HS-induced ROS generation. The levels of γH2AX were significantly increased by HS. However, these levels were significantly decreased by combining HS and EAS (*P* < 0.05, Fig. 5b,d).

### Effect of EAS on GSH synthesis

**Figure 6:**
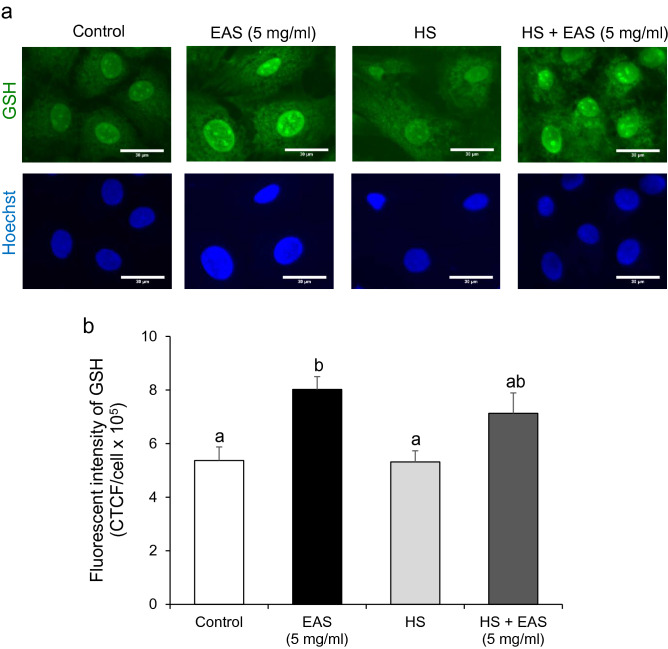
Effect of EAS on GSH synthesis in the bovine CG cells under non- and HS conditions. Cells were treated for 6 h with or without EAS (5 mg/ml) under normal conditions at 38.5 °C (control, EAS group) and HS conditions at 41 °C (HS, HS + EAS group). (**a**) Fluorescence of GSH (upper) and nuclei stained with Hoechst (lower). Scale bar shows 30 μm. (**b**) Fluorescence intensity of GSH (CTCF). Data are shown as the mean ± SEM, n = 5, a vs. b (*P* < 0.05).

GSH was detected more strongly in the nuclei than in the cytoplasm (Fig. 6a). EAS treatment significantly increased GSH levels (*P* < 0.05) in non-HS cells (Fig. 6a,b).

### Effect of EAS on expression of genes related to oxidative stress and redox balance

**Figure 7:**
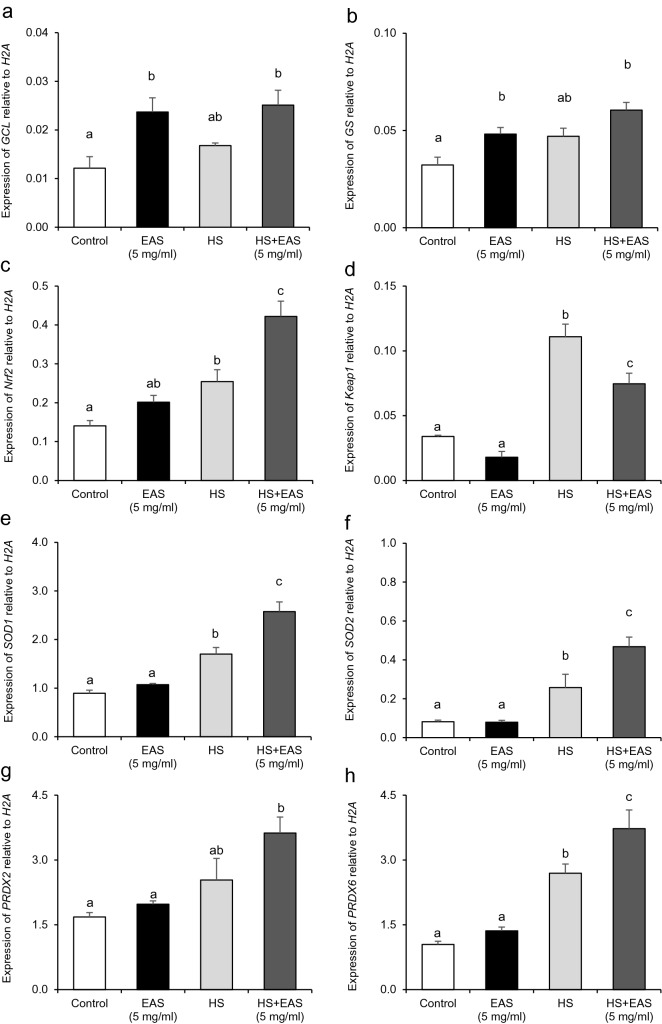
Effect of EAS on expressions of *GS, GCL, Nrf2, Keap1, SOD1, SOD2, PRDX2* and *PRDX6* under non- and HS conditions. Cells were treated for 6 h with or without EAS (5 mg/ml) under normal conditions at 38.5 °C (control, EAS group) and HS conditions at 41 °C (HS, HS + EAS group). The expression levels of *GS, GCL, Nrf2, Keap1, SOD1, SOD2, PRDX2*, and *PRDX6* were examined using real-time quantitative PCR normalized to *H2AFZ* as a reference gene. (**a**) *GCL*, (**b**) *GS*, (**c**) *Nrf2*, (**d**) *Keap1*, (**e**) *SOD1*, (**f**) *SOD2*, (**g**) *PRDX2*, (**h**) *PRDX6*. Data are shown as the mean ± SEM, n = 5; means with different letters (**a**–**c**) at each mRNA is significantly different (*P* < 0.05).

To determine whether EAS protects against DNA damage by maintaining the redox status, we focused on genes related to oxidative stress and redox balance. The expression of glutamate cysteine ligase (*GCL*) and glutathione synthetase (*GS*),, which are involved in GSH synthesis in mammalian cells^26^, was significantly increased by EAS treatment under non-HS conditions (Fig. 7a,b). Since GSH synthetic pathways, including GCL and GS activation, are dependent on nuclear factor erythroid 2-related factor2 (*Nrf2*) regulation^27^, we analyzed the expression of *Nrf2* and Kelch-like ECH-associated protein 1 (*Keap1)*. The expression of both *Nrf2* and *Keap1* was significantly increased by HS treatment (Fig. 7c,d). The *Nrf2* levels in the HS and HS + EAS (5 mg/mL) groups were significantly higher than those in the control group (*P* < 0.01). In contrast, the HS + EAS (5 mg/mL) group had a lower *Keap1* mRNA expression than the control, EAS (5 mg/mL), and HS groups (*P* < 0.01) (Fig. 7d).

EAS is extracted from *Asparagus officinalis* L., which contains high levels of antioxidants, such as carotenoids, steroidal saponins, and flavonoids, and antioxidant activity has been demonstrated in other extracts of this vegetable^28^. Therefore, we analyzed the expression of antioxidant enzymes, including superoxide dismutase (*SOD*)1, *SOD2*, peroxiredoxin (*PRDX*)2 and *PRDX6*, to evaluate the antioxidant effect of EAS under non-HS conditions. The expression of *SOD1* and *SOD2* was significantly increased by HS treatment (*P* < 0.05) (Fig. 7e,f). Although no effect of EAS was observed on *SOD1* and *SOD2* expression under non-HS conditions, EAS treatment significantly increased the expression of both *SOD1* and *SOD2* under HS conditions. Similar to the expression patterns of *SOD1* and *SOD2*, EAS treatment significantly increased the expression of *PRDX2* and *PRDX6* (*P* < 0.05) (Fig. 7g,h).

### Effect of HSP70 inhibition on the redox status of bovine CG cells treated with EAS

The high level of HSP70 reduced ROS levels and increased GSH levels during EAS treatment, suggesting that HSP70 plays a role in the balance of the cellular redox status in bovine CG cells. We hypothesized that HSP70 induction by EAS triggers the regulation of the balance between ROS and GSH levels, which is required for maintaining the cellular redox status in bovine CG cells. To test this hypothesis, cells were treated with EAS (5 mg/mL) and 10 µM pifithrin-µ (PES). PES has been reported to inhibit HSP70 by interacting with the C-terminal peptide substrate-binding domain^29^. The viability of cells treated with 10 µM PES was 77% (Supplemental Fig. 1).

**Figure 8:**
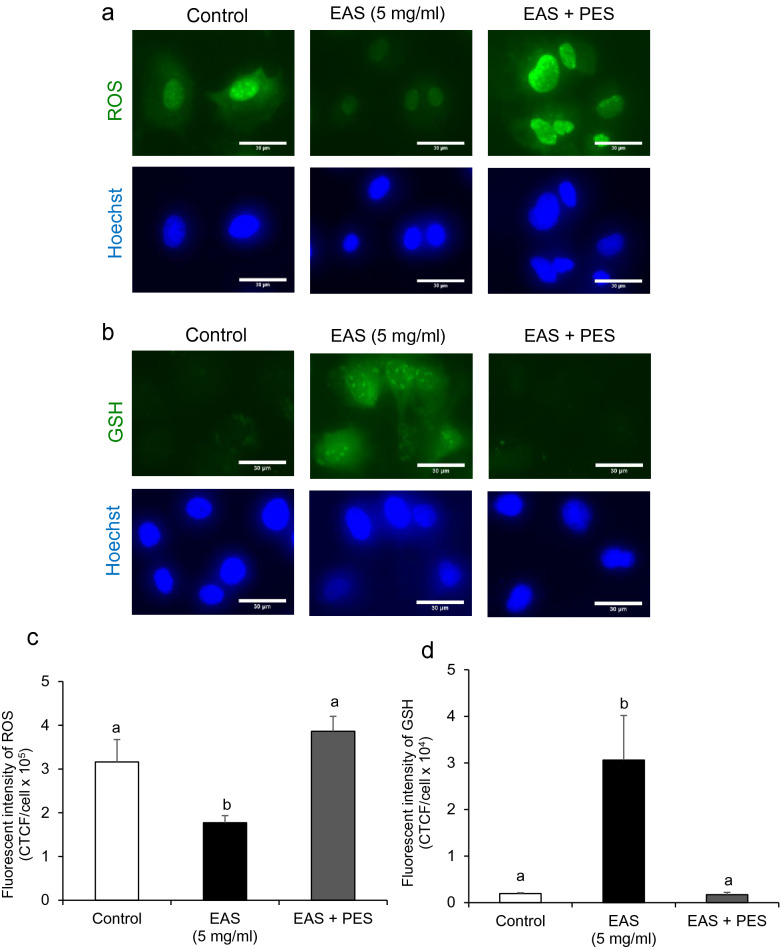
Effect of EAS-induced HSP70 inhibition on GSH synthesis and ROS generation in bovine CG cells. Cells were treated for 6 h with or without EAS (5 mg/ml) under normal conditions at 38.5 °C (control, EAS group) and 10 µM PES together with 5 mg/ml EAS (EAS + PES). (**a**) Fluorescence of ROS (upper) and nuclei stained with Hoechst (lower). (**b**) Fluorescence of GSH (upper panel) and nuclei stained with Hoechst (lower panel). Scale bar shows 30 μm. (**c**) Fluorescence intensity of ROS (CTCF). Bar shows 30 μm. (**d**) Fluorescence intensity of GSH (CTCF). (**c**) Data are shown as mean ± SEM, n = 5, a vs. b (*P* < 0.05). (**d**) Data are shown as the means ± SEM, n = 5, a vs b (*P* < 0.01).

**Figure 9:**
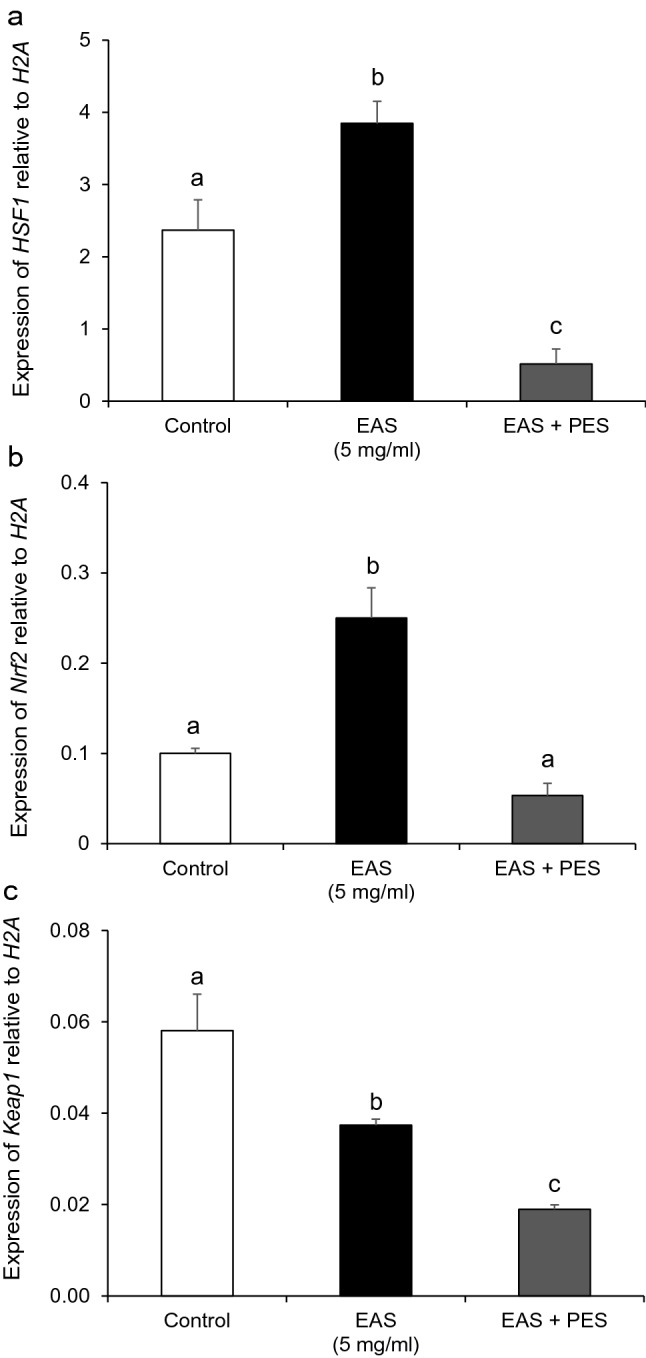
Effect of EAS-induced HSP70 inhibition on expression of *HSF1, Nrf2* and *Keap1*. Cells were treated for 12 h with or without EAS (5 mg/ml) under normal conditions at 38.5 °C (control, EAS group) and 10 µM PES together with 5 mg/ml EAS (EAS + PES). The expression levels of *HSF1, Nrf2*, and *Keap1* were examined using real-time quantitative PCR, normalized to *H2AFZ* as a reference gene. (**a**) *HSF1*, (**b**) *Nrf2*. Data are shown as the mean ± SEM, n = 5, a vs. b (*P* < 0.01), a vs. c (*P* < 0.05), and b vs. c (*P* < 0.01). (**c**) Keap1. Data are shown as the mean ± SEM, n = 5, a vs. b (*P* < 0.05), a vs. c (*P* < 0.001), and b vs. c (*P* < 0.05).

ROS levels decreased following EAS + PES treatment (Fig. 8a,c). In contrast, the levels of GSH that had been increased by EAS treatment were significantly decreased (Fig. 8b,d). Moreover, the expression of *HSF1* and *Nrf2* increased by EAS treatment was significantly decreased by the inhibition of HSP70 (Fig. 9a,b). In addition, *Keap1* expression was significantly decreased by the inhibition of HSP70 (Fig. 9c).

### Effect of EAS on progesterone synthesis

**Figure 10:**
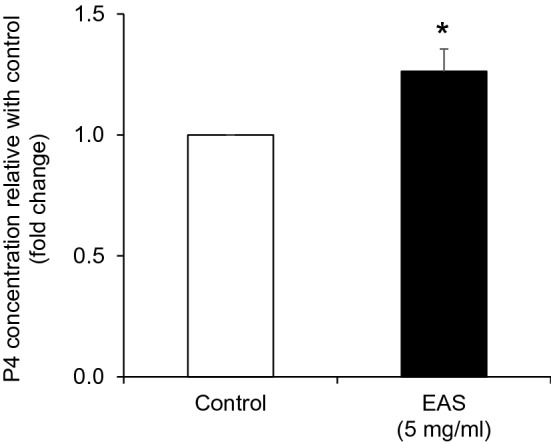
Effect of EAS on P4 synthesis in bovine CG cells. Cells were treated for 12 h with or without EAS (5 mg/ml) under normal conditions at 38.5 °C (control, EAS group). Data are shown as the mean ± SEM, n = 8, *P < 0.05, vs. control.

Bovine CG cells not only play a role in oocyte development but also perform an endocrine role of steroid hormone secretion^30^. Progesterone (P4) plays a major role in regulating pregnancy and conceptus growth in cattle^31^. We measured P4 levels to further investigate the effect of EAS treatment on steroidogenesis and found that P4 levels were significantly increased by EAS supplementation compared with that in the control group (*P* < 0.05) (Fig. 10).

## Discussion

EAS has been shown to increase the expression of HSP70 mRNA and protein in HeLa cells, neuronal cell NG108-15, and hepatocyte cells^32–34^. Moreover, *HSP70* overexpression increased the GSH/GSH disulfide ratio while reducing ROS levels under hypoxia and glucose deprivation conditions^18^. Therefore, the current study investigated whether EAS treatment increases *HSP70* expression. GSH is a tripeptide of gamma-glutamyl-cysteinylglycine with antioxidant properties, whose synthesis reduces HS-induced ROS generation with DNA damage to maintain the redox status in bovine CG cells.

In the present study, EAS at a concentration of 5 mg/mL significantly increased *HSP70* mRNA and protein levels in bovine CG cells following 6 h of incubation. This result is similar to that of a previous study, in which hepatocyte cells were treated with 4 mg/mL EAS during incubation for 4 h^34^. In addition, HeLa cells showed increased HSP70 mRNA and protein expression when treated with a lower dose of EAS (4 mg/mL) but after a longer incubation time (24 h) than that in the current study (6 h)^32^. The variation in the effect of EAS dose in cells may be because of differences in cell type and culture conditions. *HSP70* expression-enhancing activity by EAS may arise from asparagine, which has been found to elevate HSP70 expression in human monocyte HL-60 cells^20^. On the other hand, resistance to anti-cancer drugs due to the overexpression of *HSP27* and *HSP90* has been reported in several studies, but the expression of *HSP27* and *HSP90* in bovine CG cells was not affected by EAS treatment^35^. Therefore, our results confirm the unique effect of EAS on HSP70 induction in bovine CG cells under non-HS conditions.

In vivo and in vitro studies have shown a synergistic effect between HS and HSP-inducing compounds on *HSP70* expression^22,36^. A previous study showed that the maximum rectal temperature in cows was 41.1 °C during summer (heat load index > 86)^37^. In this study, HSP70 induction by EAS occurred under both non-HS and HS conditions, and it was highest under HS conditions^35^. These data suggest that the combination of EAS and HS synergistically affects HSP70 induction in bovine CG cells. The induction of HSF1, which is one of the main transcription factors of HSP70, by EAS has been reported in neuronal cells NG108-15^34^. The significant increase in *HSF1* gene expression by EAS in the present study supports the involvement of EAS in the HSP70 inducing pathway in bovine CG cells^37^. Under cellular stress, HSF1 is activated and releases HSP70 to prevent the formation of misfolded polypeptides^38^. In this study, *HSF1* gene expression was induced by EAS supplementation in both non-HS and HS conditions, and it was highest with EAS supplementation under HS conditions. These data suggest that EAS and HS have synergistic effects on the induction of HSF1 in bovine CG cells. HSF2 is a HS transcription factor co-expressed with HSF1, which is activated in response to distinct developmental cues or differentiation stimuli^38^. However, *HSF2* was not affected by EAS in the current study and supplementation with EAS led to increased HSP70 expression under non-HS conditions. However, *HSF1* expression was only induced under HS conditions, with a synergistic effect of EAS. In addition, treatment with an HSP70 inhibitor reduced *HSF1* expression. The inconsistency with results from previous studies may be due to several factors. EAS may regulate the expression of HSP70 not only through *HSF1* gene expression but also through other regulatory mechanisms. Additionally, research on different species and cell types has led to reports of different regulatory effects of EAS on *HSF1* gene expression^34^. Hence, it is conceivable that an increase in HSP70 expression by EAS is induced as a result of the asparagine content and enhancement of HSF1, with EAS exerting synergistic effects with HS on HSP70 induction.

Several studies have indicated that HSP70 has apoptosis-suppressive effects and anti-inflammatory activity, indicating that HSP70 exerts a cytoprotective function against various stresses^39^. In addition, the overexpression of HSP70 reduces ROS induced by hypoxia and glucose deprivation^18^. These results indicate that EAS with a unique inducible HSP70 could protect cells against ROS under various stress conditions. Moreover, EAS was found to significantly reduce the amyloid beta peptide-induced production of ROS in differentiated rat PC12 cells^40^. In this study, EAS treatment significantly inhibited ROS expression in both normal and HS conditions in bovine CG cells by activating GSH generation and antioxidant enzymes, such as SOD and PRDX. Moreover, ROS activates c-Jun N-terminal kinase protein, which also increases under HS conditions^41^. EAS clearly reduced the c-Jun N-terminal kinase protein induced by hydrogen peroxide in fibroblast cells^42^. In conclusion, EAS reduced HS-related ROS generation in bovine CG cells, which can be explained by the upregulation of GSH generation together with antioxidant enzymes and the downregulation of c-Jun N-terminal kinase protein.

ROS-induced damage can cause both single- and double-stranded DNA breaks^25^. In this study, EAS significantly reduced *γH2AX* levels under HS conditions. ROS generation from normal cellular metabolism and HS induces DNA damage in cells^43^. In a previous study, EAS was shown to reduce cell damage induced by nitric oxide donor sodium nitroprusside or the hypoxia mimic reagent cobalt chloride of NG108-15 cells^33^. HSP70 has the ability to repair DNA damage caused by HS^25^. In this study, HSP70 induction by the synergistic effect of EAS and HS contributed to the reduction of DNA damage in bovine CG cells supplemented with EAS under HS conditions. Moreover, GSH also contributes to DNA repair activity, and the expression of GSH in the nucleus enhances protection against DNA damage^44^. In the present study, GSH generation was increased by EAS in bovine CG cells under non-stress and HS conditions. These results indicate that EAS reduced DNA damage under non-HS conditions and was within the acceptable range of non-toxic levels of ROS production due to the enhancement of HSP70 and GSH levels.

In the present study, EAS increased GSH in both non-HS and HS conditions, but only cells supplemented with EAS under non-HS conditions were significantly different when compared with the control group. In agreement with this observation, previous reports have suggested that overexpression of HSP70 enhances GSH expression^18^. However, the GSH levels in the control group were similar to those in the HS group, which were exposed for 6 h at 41 °C. In HeLa cells, HS treatment for 1, 2, or 3 h at 42 °C resulted in the highest increase in GSH level at 1 h and the lowest increase at 3 h^27^. GSH biosynthesis is required by the action of two ATP-dependent enzymes: GCL, which assembles the formation of c-glutamyl-cysteine from glutamate-cysteine, and GS, which is involved in the ligation of c-glutamyl-cysteine to glycine in another ATP-dependent reaction to yield GSH^26^. Our results showed that EAS increased the expression of GCL and *GS* under non-HS conditions. The transcription factor Nrf2, which has the potential to induce GCL and GS, is activated by ROS production^45^. Moreover, HSF1 may induce Nrf2 by activating p62, which can separate Nrf2 from Keap1^46^. In this study, GSH levels were not decreased by HS because of the compensatory effect of HSF1 with Nrf2, which can influence GSH levels after 6 h of HS treatment. Therefore, there is a need to study the effect of HS treatment on GSH synthesis and the induction of genes. A previous study showed that reduced Keap1 expression is logically related to the induction of Nrf2^47^. Together with the induction of *Nrf2*, EAS supplementation reduced *Keap1* and induced *HSF1* expression in this study. EAS treatment increased Nrf2 protein levels in NG108-15 neuronal cells^33^. In conclusion, EAS increased GSH expression and γ-glutamyl cycle mRNA expression due to Nrf2, which was induced by the compensation effect of HSF1 with Nrf2 and the antioxidant content of EAS.

The higher expression of antioxidant enzyme genes in the HS group in the present study was similar to that reported in a previous study, in which HS induced SOD and PRDX expression in pig skeletal muscle^48^ and HeLa cells^27^, respectively. In another study, HS was reported to upregulate HSP70 and SOD2 in Chinese hamster lung fibroblast V79 cells^41^. Interestingly, the HSP family and antioxidant system reduced the harmful effects of ROS^49^. In this study, EAS increased *HSP70* expression, antioxidant enzymes, and reduced ROS levels. Nrf2 expression has been shown to induce PRDX activity in HeLa cells^26^ and SOD activity in mesenchymal stem cells^50^. In this study, Nrf2 expression was highest in bovine CG cells treated with EAS under HS conditions, indicating that the induction of *PRDX* and *SOD* was due to *Nrf2* expression. EAS extracted from asparagus (*Asparagus officinalis* L.) contains high levels of antioxidants, including carotenoids, steroidal saponins, and flavonoids^28^. Therefore, the enhanced antioxidant activity of EAS treatment results from the induction of Nrf2 expression and the antioxidant content of asparagus (*Asparagus officinalis* L.).

The superoxide dismutase-like activity of *Trapa japonica* shell extract was lower than that of ascorbic acid^51^. In addition, the 1,1-diphenyl-2-picryl hydrazyl radical scavenging activity of saffron petals, a by-product of saffron, was also significantly lower than that of ascorbic acid^52^. Thus, the improvement of antioxidant activity by EAS supplementation is not caused by the antioxidant components contained in EAS, but by EAS itself.

PES has been shown to inhibit HSP70^29^. In this study, PES reversed the effect of EAS on GSH and ROS levels, indicating that HSP70 induction by EAS regulated the levels of GSH and ROS in bovine CG cells. A previous study reported a correlation between the transcription factors Nrf2 and HSF1 for the protection of cells^53^. There is evidence that Nrf2 and HSF1 compensate each other; the induction of HSP70 by methionine deprivation is dependent on Nrf2 but independent of HSF1^54^. Both Nrf2 and HSF1 play important roles in cellular redox processes because of their ability to influence the levels of HSP70 and GSH. Therefore, distinct cell survival pathways, such as the HS response and Keap1/Nrf2/ARE pathway, are regulated by Nrf2 and HSF1^55^. In the present study, PES also reversed the induction effect of both *Nrf2* and *HSF1*. PES reduces the nuclear translocation of the nuclear factor-κB (NF-κB) pathway, which regulates the transcription of various gene families including: stress response, apoptosis, and receptor genes, and influences cell survival, differentiation, and proliferation^56^. Nuclear translocation of NF-κB p65 enhances the ability of Nrf2 and plays a role in the antioxidant response in human kidney-2 cells^57^. In addition, HSF1 activation in intestinal epithelial cells during HS is regulated by the NF-κB pathway^58^. Moreover, PES reduced the levels of Keap1, which combined with Nrf2 to regulate the antioxidative protection system^55^. These results indicate that HSP70 induction by EAS improved the redox balance by regulating the ROS and GSH levels through HSF1, and Nrf2/Keap1 pathway in bovine CG cells.

The beneficial effect of EAS on P4 synthesis demonstrated in the study was similar to that reported in a previous study, in which the oral administration of asparagus root extract enhanced P4 levels in rats^59^. This indicates that bovine CG cells are important for reproductive functions, especially steroidogenesis. These findings indicate that EAS contributes to the improvement of P4 synthesis in bovine CG cells.

In conclusion, EAS was shown to induce HSP70 under non-HS conditions, exerting a synergistic effect with HS on HSP70 induction in bovine CG cells. Furthermore, EAS had beneficial effects, reducing the DNA damage induced by ROS, and increasing GSH synthesis and antioxidant enzyme levels to maintain the redox status, in addition to the P4 levels in bovine CG cells. HSP70 induced by EAS regulated the Nrf2/Keap1 pathway and HSF1 transcription factor levels, which contributed to the ROS and GSH levels in bovine CG cells.

The findings presented in this study collectively demonstrate that EAS has potential uses in the regulation of reproductive functions by reducing physical stress and improving the properties of reproductive cells.

## Methods

EAS, which is produced from asparagus (*A. officinalis* L.) grown in Hokkaido, was provided by Amino Up Co., Ltd. (Sapporo, Japan). EAS was manufactured according to a previously described method^60^.

### Bovine CG cell culture

Bovine ovaries were collected from a local abattoir and transported to the laboratory at 20 °C. The ovaries were washed several times with sterile saline solution. Follicular contents were aspirated from follicles (2–8 mm in diameter), which lacked evident signs of atresia using a disposable 18-gauge needle attached to a 10-ml syringe. These follicular contents were released into a sterile plastic Petri dish, and cumulus-oocyte complexes were picked up. After picking up the cumulus-oocyte complexes, the suspension of follicular fluid with floating CG cells was cultured in 5% fetal bovine serum (FBS) in Dulbecco’s modified Eagle’s medium (high glucose) (DMEM) (Wako, Osaka, Japan) supplemented with 0.06 g/l penicillin G potassium (Nacalai Tesque, Kyoto, Japan) and 0.1 g/L streptomycin sulfate (Nacalai Tesque) at 38.5 °C under 5% CO_2_ in air. After overnight incubation, a network of theca cell clusters was gently peeled from the culture dish surface. Bovine CG cells remaining in the culture dish were washed with calcium- and magnesium-free phosphate-buffered saline (PBS) (–) and cultured in 5% FBS in DMEM at 38.5 °C under 5% CO_2_ in air. After reaching confluency, bovine CG cells were washed with PBS (–) and dissociated from the substratum with PBS (–) containing 0.05% trypsin and 0.53 mM EDTA for 2 min at 38.5 °C in a CO_2_ incubator. After supplementation with 5% FBS in DMEM to inhibit trypsin activity, the cell suspension was centrifuged at 1,200 × *g* for 3 min.

Viable cells were plated at a density of 1.0 × 10^5^ cells/mL onto 4-well culture plates (Thermo Fisher Scientific) or 8-well slides and chambers (SPL Life Sciences Co., Ltd, Pocheon, Korea) and cultured at 38.5 °C under 5% CO_2_ in air. After cells became 70% confluent, the medium was replaced with 0.9 ml of 5% FBS in DMEM together with 0.1 ml of EAS stock solution in PBS (–), and 0.1 ml of PBS (–) was added in control group. The cells were then cultured at 38.5 °C or at 41 °C under 5% CO_2_ in air under HS conditions.

### Experimental design

Cells were cultured at 38.5 °C for 6 h with 0.5, 1, and 5 mg/mL of EAS for mRNA expression of *HSP27*, *HSP90* and, *HSP70.*To compare the effect of EAS under non-HS and HS conditions, cells were cultured at 38.5 °C and 41 °C for 6 h with 5 mg/mL of EAS for western blotting, immunostaining, fluorescent staining and mRNA expression.

To inhibit HSP70 activity, cells with 5 mg/mL of EAS supplementation were treated with or without 10 µM PES under non-HS conditions for 12 h.

To determine the optimal concentration of PES, cells were treated with 5 µM, 10 µM, or 20 µM PES under non-HS conditions for 12 h and cell viability was analyzed using the Live-Dead Cell Staining Kit (ALX-850–249, Enzo Life Sciences AG, Lausen, TX, USA) according to the manufacturer’s instructions.

To determine the influence of EAS on P4 synthesis, CG cells were cultured at 38.5 °C for 12 h with 5 mg/mL of EAS.

### RNA extraction and quantitative reverse-transcription polymerase chain reaction (RT-qPCR)

The cells were collected with the ISOGEN II. All RNA samples were stored at − 80 °C. RNA concentration was measured using spectrophotometry (NanoDrop ND-2000; Thermo Fisher Scientific). Complementary DNA was synthesized by reverse transcription using the ReverTra Ace qPCR RT Master Mix with gDNA remover (Toyobo Life Science, Osaka, Japan) according to the manufacturer’s instructions using a thermal cycler (Astec GeneAtlas Type G Thermal Cycler; ASTEC, Fukuoka, Japan). All cDNA samples were stored at − 30 °C. Specific primers (Supplementary Table 1) were designed using Primer-BLAST (http://www.ncbi.nlm.nih.gov/tools/primer-blast/). The relative expression levels were assessed via qRT-PCR using a LightCycler Nano (Roche Diagnostics, Basel, Switzerland) and THUNDERBIRD SYBR qPCR Mix (Toyobo Life Science) at a final primer concentration of 0.5 μM for each primer. The thermal cycling conditions were as follows: 1 cycle at 95 °C for 30 s (denaturation), 45 cycles at 95 °C for 10 s (denaturation), 55 °C for 15 s (primer annealing), and 72 °C for 30 s (extension). Relative mRNA abundance was calculated using the ΔΔCt method, with *H2AFZ* as a reference gene.

### Western blotting

Bovine CG cells were lysed in 1% SDS (Nacalai Tesque), 1% 2-mercaptoethanol (Nacalai Tesque), 20% glycerol (Nacalai Tesque), and 50 mM Tris–HCl (pH 6.8) and denatured at 95° C for 5 min. Sample solutions were separated by electrophoresis on 10%–20% gradient SDS–polyacrylamide precast gels (Atto Corporation, Tokyo, Japan). Pre-stained marker proteins with known BlueStar (range, 10–180 kDa) (cat. no. MWP03; Nippon Genetics Co., Ltd., Tokyo, Japan) were run as standards. The electrophoretically separated proteins were transferred onto PVDF membranes using an iBlot Gel Transfer System (Invitrogen, Thermo Fisher Scientific Inc., Massachusetts, USA). The membranes were incubated in 4% skim milk (FUJIFILM Wako Pure Chemical Corporation, Osaka, Japan) for 10 min and then washed three times with Tris-buffered saline and Tween-20 (TBS-T) at room temperature. Membranes were incubated with rabbit anti-human HSP70 polyclonal (1:1,000 dilution) (SPC-103; StressMarq Biosciences Inc.) and β-Actin monoclonal (1:1,000 dilution; cat. no. 66009–1-Ig; Proteintech Group, Rosemont, USA) at 4 °C overnight. After three washes with TBS-T, the membranes were incubated with HRP-labeled anti-rabbit IgG for HSP70 (1:25,000 dilution; cat. no. NA934; GE Healthcare, Buckinghamshire, UK) or anti-mouse IgG secondary antibody for β-actin (1:25,000 dilution; cat. no. NA931; GE Healthcare, Buckinghamshire, UK) at room temperature for 1 h. The primary and secondary antibodies were diluted with an immunoreaction enhancer, Can Get Signal (Toyobo). Membranes were washed with TBS-T before detection of bound antibodies using the WSE-7120EzWestLumi plus (Atto Corporation, Tokyo, Japan). Chemiluminescent signals were captured using LumiCube (Liponics, Inc., Tokyo, Japan). The intensity of the bands was analyzed using ImageJ software v1.52A (National Institutes of Health; http://imagej.nih.gov/ij/).

### Immunostaining for HSP70 and γH2AX

Bovine CG cells were fixed in 4% paraformaldehyde diluted with PBS (–) for 15 min. After washing the cells three times with PBS (–) for 5 min, the samples were permeabilized with PBS (–) containing 0.2% (v/v) Triton X-100 for 10 min. After washing the cells with PBS (–), they were blocked with 2% (w/v) BSA (Sigma-Aldrich) in PBS (–) for 1 h at room temperature. The samples were washed with PBS (–), incubation with a Rabbit Anti-Human HSP70 Polyclonal (SPC-103; Stress Marq Biosciences Inc.) diluted 1:500 with 0.1% (w/v) BSA (Sigma-Andrich) or primary rabbit polyclonal antibody for γH2AX (ab11174; Abcam, Cambridge, MA, USA) diluted 1: 1,000 in PBS (–) at 4 °C overnight. The samples were each washed three times with PBS (–) for 5 min and incubated for 1 h with a fluorescein-conjugated secondary antibody (Alexa Fluor 488 donkey anti-rabbit IgG) (A21206; Thermo Fisher Scientific) diluted 1:500 for HSP70 or 1:1,000 for γH2AX with 0.1% (w/v) BSA (Sigma-Aldrich) in PBS (–) at room temperature. Cells were then washed with PBS (–) for 5 min, and 10 μL of the mounting solution (Fluoro-KEEPER Anti Fade Reagent) (Non-Hardening Type with DAPI; Nacalai Tesque) was added to the samples. The samples were then covered with a cover glass. Fluorescence images were acquired using an EVOS M5000 Imaging System (Invitrogen, Thermo Fisher Scientific Inc., Massachusetts, USA). Fluorescence intensity was quantified using ImageJ software.

### Detection of ROS and GSH

Cells were washed with PBS (–) and treated with 5 μM CellROX Oxidative Stress Reagents (cat. no. C10444; Life Technologies, Carlsbad, CA, USA) for ROS staining, or 20 µM ThiolTracker Violet (cat. no. T10095, Molecular Probes, Eugene, OR, USA) for GSH staining.

Fluorescence images were acquired using an EVOS™ M5000 imaging system (Thermo Fisher Scientific Inc.). Fluorescence intensity was quantified using ImageJ software v1.52A (National Institutes of Health; http://imagej.nih.gov/ij/). For corrected total cell fluorescence (CTCF), we used the following formulas^61^:

1. CTCF = integrated density (total area of selected cells × mean fluorescence of background readings)
2. CTCF per cell = CTCF/ Ncells

where “Integrated Density” is the integrated intensity of the pixels for all cells in the image, total cell area is the number of pixels of all of the cells, background fluorescence is the average mean gray value of nearby regions containing no cells, and Ncells is the number of cells that was measured by counting fluorescently labeled nuclei from images.

### Measurement of progesterone

The culture medium was collected and centrifuged. The supernatant of the culture medium was used to measure progesterone through enzyme-linked immunosorbent assay using commercial kits following manufacturer’s instructions (ADI- 900–011, Enzo Life Sciences, Inc., USA).

### Statistical analysis

All data are shown as the mean ± standard error of the mean (SEM). Analysis of variance (ANOVA), Tukey’s test, and Student’s t-test were performed using R (version 3.5.3; https://www.r-project.org/). Statistical significance was set at *P* < 0.05.

## Supplementary Information

Supplementary Information 1.

Supplementary Information 2.

Supplementary Legends.

Supplementary Information 4.

## Data Availability

All study data are included in this article with supplementary files.
