# Supplementary material for: A standardized extract of Asparagus officinalis stem improves HSP70-mediated redox balance and cell functions in bovine cumulus-granulosa cells
Source: Sci Rep. 2021 Sep 13;11:18175. doi: 10.1038/s41598-021-97632-6 (PMC8437968; doi:10.1038/s41598-021-97632-6)
Supplement: Supplementary file 1 — Supplementary Information 1. [file 41598_2021_97632_MOESM1_ESM.pptx]

## Slide 1
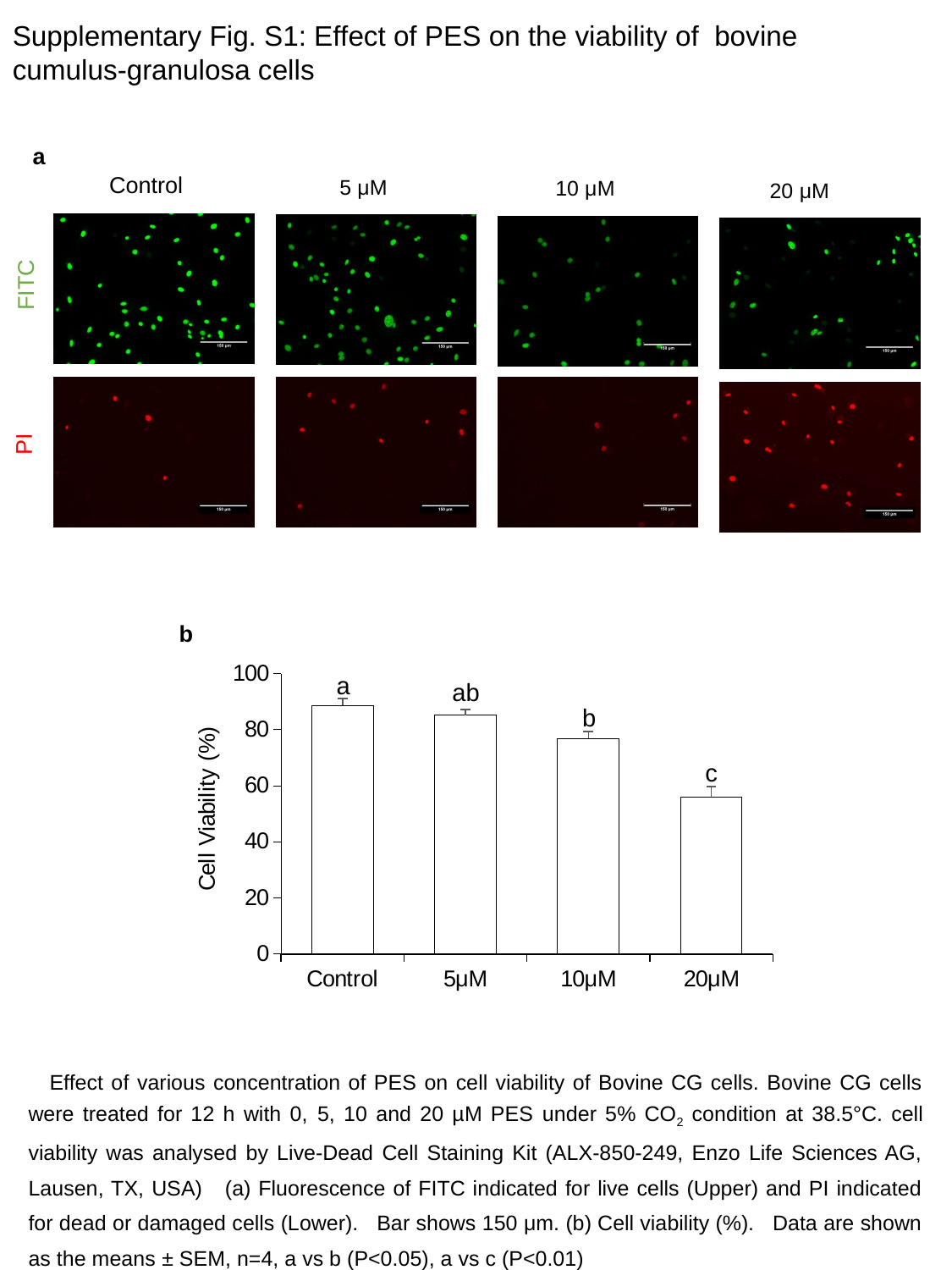

Supplementary Fig. S1: Effect of PES on the viability of bovine cumulus-granulosa cells
a
Control
5 μM
10 μM
20 μM
FITC
PI
b
### Chart
| Category | |
|---|---|
| Control | 88.58720827203081 |
| 5μM | 85.11027156537992 |
| 10μM | 76.81422919065757 |
| 20μM | 55.97038815526211 |a
ab
b
c
Effect of various concentration of PES on cell viability of Bovine CG cells. Bovine CG cells were treated for 12 h with 0, 5, 10 and 20 µM PES under 5% CO2 condition at 38.5°C. cell viability was analysed by Live-Dead Cell Staining Kit (ALX-850-249, Enzo Life Sciences AG, Lausen, TX, USA) (a) Fluorescence of FITC indicated for live cells (Upper) and PI indicated for dead or damaged cells (Lower). Bar shows 150 μm. (b) Cell viability (%). Data are shown as the means ± SEM, n=4, a vs b (P<0.05), a vs c (P<0.01)
