# Supplementary material for: A standardized extract of Asparagus officinalis stem improves HSP70-mediated redox balance and cell functions in bovine cumulus-granulosa cells
Source: Sci Rep. 2021 Sep 13;11:18175. doi: 10.1038/s41598-021-97632-6 (PMC8437968; doi:10.1038/s41598-021-97632-6)
Supplement: Supplementary file 2 — Supplementary Information 2. [file 41598_2021_97632_MOESM2_ESM.docx]

Suppl Table 1. Primer sequences used for qRT-PCR

| Accession No. | Gene | Forward (5'-3') | Reverse (5'-3') |
| --- | --- | --- | --- |
| NM_203322.3 | *HSP 70* | GCAGTCGGACATGAAGGAGT | GATCTCCTCCGGGTAGAACG |
| NM_001012670.2 | *HSP 90* | GTATGGACAATGACTCCAATCAAGT | CCGTTTGTTGTAAGGTGTGTATGTA |
| NM_001046570.2 | *HSP27* | AAGACTGCAGGCTGGATCAC | CAGGACTTGGAAGCGGGATT |
| NM_001076809.1 | *HSF1* | TTCAAGCACAGCAACATGGC | CAGAGTGGACACACTGGTCA |
| NM_001083405.1 | *HSF2* | GTGCAGATGAATCCCACAGA | AGGGTTCCCATCGAGGAATG |
| NM_001083674.1 | *GCL* | CATTTGCAAAGGTGGCAACG | GTAGGAGTTCAGGACGGGGA |
| NM_001015630.1 | *GS* | GAGGCCAGAGTAAGGAACGC | GAGGTCCTCAGCAACACACC |
| NM_001011678.2 | *Nrf2* | ATGATGGACTTGGAGCTGCCG | TGCTCCTTCTGTCGTTGACTG |
| NM_001101142.1 | *Keap1* | AGGCTAGAGTGGGAACTCGT | CCAGGCCTAGTCTTGGGGTA |
| NM_174615.2 | *SOD1* | ACACAAGGCTGTACCAGTGC | TGTCACATTGCCCAGGTCTC |
| NM_201527.2 | *SOD2* | GGATCCCCTGCAAGGAACAA | TGGCCTTCAGATAATCGGGC |
| NM_174763.2 | *PRDX2* | ATGGTGCCTTCAAGGAGGTG | GGTGGGTGAACTGAGAGTCG |
| NM_174643.1 | *PRDX6* | CTCCTCTTACTTCCCGCGTC | GAATGCCCCATGAGTCTCCC |
| NM_174809 | *H2AFZ* | AGAGCCGGTTTGCAGTTCCCG | TACTCCAGGATGGCTGCGCTGT |
