# Supplementary material for: A standardized extract of Asparagus officinalis stem improves HSP70-mediated redox balance and cell functions in bovine cumulus-granulosa cells
Source: Sci Rep. 2021 Sep 13;11:18175. doi: 10.1038/s41598-021-97632-6 (PMC8437968; doi:10.1038/s41598-021-97632-6)
Supplement: Supplementary file 3 — Supplementary Legends. [file 41598_2021_97632_MOESM3_ESM.docx]

Supplementary Figure 2

Western blotting was performed to determine the protein expression of HSP70. Cells were treated for 6 h with or without EAS (5 mg/ml) under control at 38.5°C (control, EAS) (second and third lanes) and HS conditions at 41°C (HS, HS + EAS).

In the WB gel image, left lane shows the pre-stained marker proteins (range, 10–180 kDa: cat. no. MWP03; Nippon Genetics Co., Ltd., Tokyo, Japan). To make clarify the band location, scale marker was also used for the acquisition of chemiluminescent image.

HSP70 polyclonal antibody(1:1,000 dilution) (SPC-103; StressMarq Biosciences Inc.) (70 kDa) and β-Actin monoclonal antibody (1:1,000 dilution; cat. no. 66009-1-Ig; Proteintech Group, Rosemont, USA) (42 kDa) were used for the detection of the expression of HSP70 and β-Actin, respectively. Chemiluminescent signals were captured using LumiCube (Liponics, Inc., Tokyo, Japan) after 30 seconds exposure time.

Second and third lanes indicate control and EAS treatment groups cultured at 38.5°C, respectively. Fourth and fifth lanes indicate the HS and EAS treatment groups cultured at 41°C, respectively.
