# Supplementary figures and images for: A standardized extract of Asparagus officinalis stem improves HSP70-mediated redox balance and cell functions in bovine cumulus-granulosa cells
Source: Sci Rep. 2021 Sep 13;11:18175. doi: 10.1038/s41598-021-97632-6 (PMC8437968; doi:10.1038/s41598-021-97632-6)

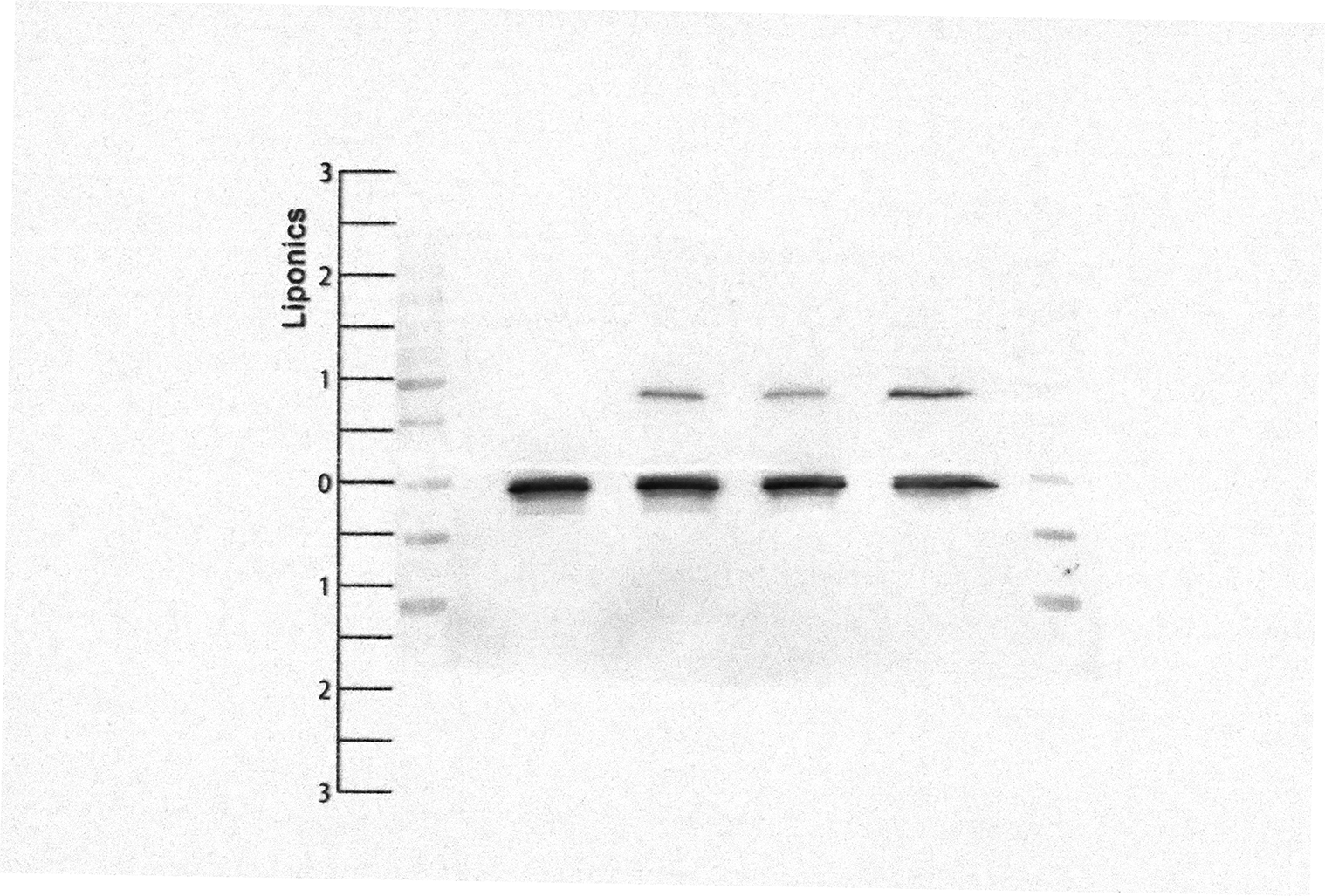

Supplement: Supplementary file 4 — Supplementary Information 4. [file 41598_2021_97632_MOESM4_ESM.tif]
